# Effects of Tai Chi in Patients with Chronic Obstructive Pulmonary Disease: Preliminary Evidence

**DOI:** 10.1371/journal.pone.0061806

**Published:** 2013-04-23

**Authors:** Jun-Hong Yan, Yong-Zhong Guo, Hong-Mei Yao, Lei Pan

**Affiliations:** 1 Department of Clinical Medical Technology, Affiliated Hospital of Binzhou Medical University, Binzhou, PR China; 2 Department of Respiratory Medicine, Central Hospital of Xuzhou, Affiliated Xuzhou Hospital of Medical College of Southeast University, Xuzhou, PR China; 3 Department of Internal Medicine, State Key Laboratory of Respiratory Disease, First Affiliated Hospital, Guangzhou Medical University, Guangzhou, PR China; Università Vita-Salute San Raffaele, Italy

## Abstract

**Background:**

Currently, several studies assessed the role of Tai Chi (TC) in management of chronic obstructive pulmonary disease, but these studies have wide variation of sample and convey inconclusive results. We therefore undertook a meta-analysis to assess the effects of TC.

**Methods:**

A computerized search through electronic databases was performed to obtain sample studies. The primary outcomes were 6-min walking distance (6MWD) and dyspnea. Secondary outcomes included health-related quality of life and pre-bronchodilator spirometry. Weighted mean differences (WMDs) and 95% confidence intervals (CIs) were calculated and heterogeneity was assessed with the I^2^ test. A random-effects meta-analysis model was applied.

**Results:**

Eight randomized controlled trials involving 544 patients met the inclusion criteria. The pooled WMDs were 34.22 m (95% CI 21.25–47.20, P<0.00001) for 6 MWD, –0.86 units (95% CI –1.44––0.28, P* = *0.004) for dyspnea, 70 ml (95% CI 0.02–0.13, P* = *0.01) for FEV_1_, 120 ml (95% CI 0.00–0.23, P* = *0.04) for FVC. TC significantly improved the Chronic Respiratory Disease Questionnaire total score, and the St George’s Respiratory Questionnaire score except impact score.

**Conclusions:**

Findings suggest that TC may provide an effective alternative means to achieve results similar to those reported following participation in pulmonary rehabilitation programs. Further studies are needed to substantiate the preliminary findings and investigate the long-term effects of TC.

## Introduction

Chronic obstructive pulmonary disease (COPD) is an important cause of morbidity and mortality worldwide [1]–[3]. Quadriceps weakness, decline of health-related quality of life (HRQoL) and exacerbations of symptoms such as dyspnea and cough with or without sputum production may contribute to the severity of COPD in individual patients [3]–[5]. At present, the primary goals in the management of COPD are to alleviate symptoms, slow down the deterioration of lung function, maintain or improve exercise capacity and HRQoL, which minimize the disabling effects and maximize the benefits for COPD patients [3]. The recent evidence-based clinical practice guidelines and statements showed that pulmonary rehabilitation programs (PRPs) are widely accepted as the most effective non-pharmacotherapy in the management of COPD [3], [6]–[10]. However, out-patient of PRPs are still not widely available and even those who participated in a PRP show poor adherence [11].

Tai Chi (TC), developed from ancient China as a defensive martial art, is a mind-body practice rooted in Chinese philosophies. TC provides mild to moderate aerobic activity, and lower-extremity, unsupported upper-extremity muscle strength training [12]. In recent decades, TC has gained popularity in Western countries as an alternative form of exercise. Studies have found positive effects of TC on balance control, cardiovascular fitness, pain, and fatigue, as well as effects on psychological well-being including enhanced mood and reduction of stress, anxiety and depression in both healthy participants and patients with chronic conditions [13]–[17]. In addition, TC can regulate breathing, strengthen the upper and lower limbs function, and especially can improve respiratory muscle and quadriceps muscle strength, which are essential aspects of COPD management [18]. Nowadays, there are published randomized controlled trials (RCTs) regarding the effect of TC in COPD patients [12], [19]–[21]. However, these studies have wide variation in sample size and different measures to evaluate outcomes, and convey inconclusive results. Therefore, we performed a meta-analysis to assess the effectiveness of TC as a method of PRP for COPD patients.

### Study Characteristic, Quality and Bias Assessment

## Methods

### Data Sources and Searches

A computerized search was performed through PubMed, Embase, CNKI (China National Knowledge Infrastructure) databases and other websites (e.g., Cochrane Central Register of Controlled Trials, Google Scholar, and ClinicalTrials.gov) (up to Nov 2012), were searched for original research articles using the key terms “Tai Chi” and “COPD”. Results were limited to studies with human subjects and randomized controlled trials. No language restriction was imposed. Bibliographies of all potentially relevant retrieved studies, identified relevant articles (including unpublished and meta-analysis studies, a follow-up from reference lists of relevant articles and personal contact with experts in this field) and international guidelines were searched by hand.

The following inclusive selection criteria in PICOS order included: (i) population: patients with COPD; (ii) intervention: Tai Chi or Tai Chi Qigong with or without other treatments; (iii) comparison intervention: any type of control; and (iv) outcome measures: the primary outcome measures were 6-minute walking distance (6 MWD) and dyspnea, and secondary outcomes included HRQoL and pre-bronchodilator spirometry such as forced expiratory volume in one second (FEV_1_) and forced vital capacity (FVC); and (v) study design: RCT.

In our study, dyspnea was evaluated by Borg scale and higher Borg score indicates worse dyspnea. HRQoL was evaluated by Chronic Respiratory Disease Questionnaire (CRDQ) and St George’s Respiratory Questionnaire (SGRQ). The higher CRDQ score indicates more favorable while a lower SGRQ score indicates more favorable.

### Data Extraction and Quality Assessment

For each study, we recorded the first author, year of publication, the sample size of the study population (intervention/control), type of TC protocol, study design, intervention group (TC with or without other treatments), control group (any type of other treatments), intervention duration and frequency, exercise time, and outcomes including intergroup differences. To assess eligibility, data and trial quality information from the papers selected for inclusion in the meta-analysis were extracted independently by two investigators (JHY and YZG). Any disagreements were resolved by discussion and consensus. A third investigator (HMY) was consulted in case of disagreement to improve accuracy. The analytical data missing from the primary reports were requested from their authors. When the same population was reported in several publications, we retained only the most informative article or complete study to avoid duplication of information.

### Quality Assessment and Risk-of-bias Assessment

The methodological quality of each study was evaluated using the Jadad scale [22]. A score ≤2 indicates low quality and a score ≥3 indicates high quality [23]. The risk of bias was assessed using the *Cochrane Handbook for Systematic Reviews of Interventions* (Revman version 5.1.0, The Cochrane Collaboration 2011). Two authors (JHY and YZG) subjectively reviewed all studies and assigned a value of ‘high’, ‘low’, or ‘unclear’ to the following: (a) selection bias (Was there adequate generation of the randomization sequence? Was allocation concealment satisfactory?); (b) blinding (i.e., performance bias and detection bias) (Was there blinding of participants, personnel, and outcome assessment?); (c) attrition bias (Were incomplete outcome data sufficiently assessed and dealt with?); (d) reporting bias (Was there evidence of selective outcome reporting?); and (e) other biases (Was the study apparently free of other problems that could put it at a high risk of bias?).

### Statistical Analysis

All data were combined using Revman 5.1.0. For continuous outcomes, a mean difference was calculated using weighted mean difference (WMD). All measures were estimated from each study with the associated 95% confidence intervals (CIs) and pooled across studies using a random-effects model [24]. Heterogeneity across studies was tested by using the I^2^ statistic, which was a quantitative measure of inconsistency across studies. Studies with an I^2^ statistic of 25% to 50% were considered to have low heterogeneity, those with an I^2^ statistic of 50% to 75% were considered to have moderate heterogeneity, and those with an I^2^ statistic of >75% were considered to have a high degree of heterogeneity [25]. If I^2^>50%, potential sources of heterogeneity were identified by sensitivity analyses conducted by omitting one study in each turn and investigating the influence of a single study on the overall pooled estimate. Potential publication bias for each analysis was assessed visually using a funnel plot. However, publication bias was not assessed because of the limited number (below 10) of studies included in each analysis. We undertook sensitivity analyses to explore the influence of the total effect for the primary outcomes according to methodological quality. A P value <0.05 was considered statistically significant. An intention-to-treat (ITT) analysis was applied and the overall treatment effect was compared with its minimum clinically important difference (MCID). Furthermore, this study followed the Preferred Reporting Items for Systematic Reviews and Meta-Analyses (PRISMA) statement [26].

## Results

### Bibliographic Search Results

**Figure 1:**
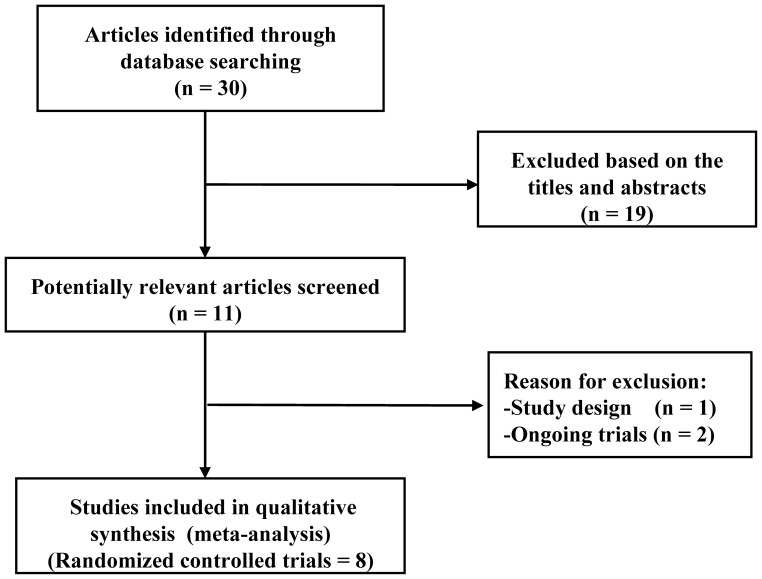
Search strategy and flow chart of screened, excluded, and eventually analyzed articles.

The initial search yielded 30 relevant publications, among which 22 were excluded for various reasons based on the inclusion criteria (PICOS). Reasons for exclusion are presented in Figure 1. Finally, eight RCTs [12], [19]–[21], [27]–[30] were selected for this meta-analysis and two of them from the same population or trial were pooled in our meta-analysis because some important outcomes were separately included in the two RCTs [19], [20]. Four RCTs were published in English and four in Chinese [27]–[30]. In addition, two ongoing RCTs (NCT01551953 and NCT01259245) were located from ClinicalTrials.gov.

**Figure 2:**
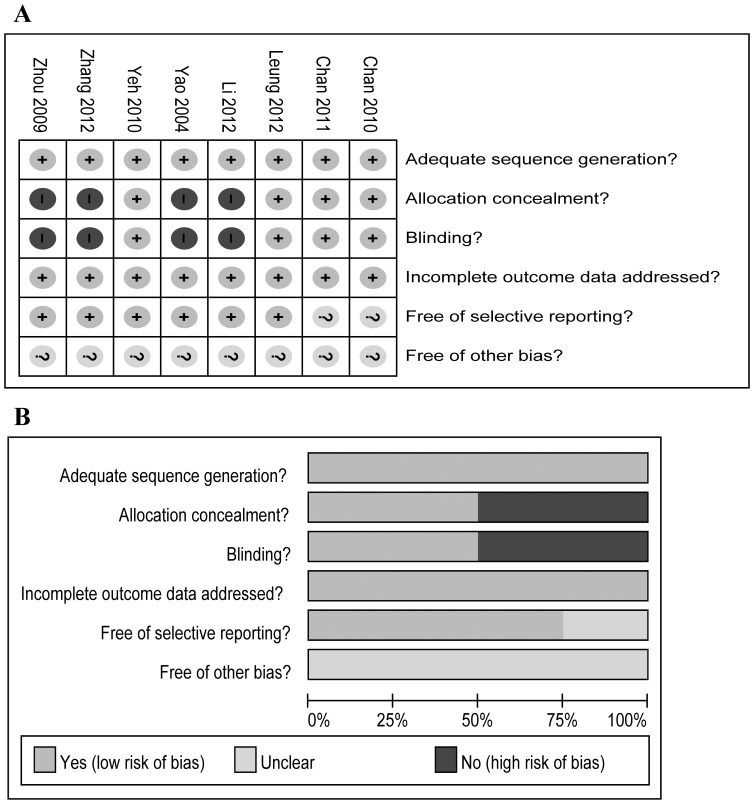
Risk-of-bias analysis. (A) Risk-of-bias summary: the authors’ judgments about each risk-of-bias item for the each included studies. (B) Risk-of-bias graph: the authors’ judgments about each risk-of-bias item presented as percentages across all included studies.

The principal characteristics of the selected studies are presented in Table 1. These studies were published between 2004 and 2012. The sample size ranged from 10 to 206 (total 544). Three RCTs reported 6 MWD [12], [19], [29] and dyspnea [19], [21], [28]. HRQoL was evaluated by CRDQ for two RCTs [12], [21] and by SGRQ for three RCTs [20], [27], [29]. Four RCTs [19], [27], [29], [30] reported FEV_1_ and three RCTs [27], [30] reported FVC. Follow-up ranged from 12 to 48 weeks and exercise time lasted 30–60 min. Two investigators (JHY and YZG) agreed on every item of the Jadad scores. The mean Jadad score of the studies included was 3 (SD = 1). Risk of bias analysis is presented in Figure 2. Four RCTs from China were not supplied with adequate allocation concealment and blinding [27]–[30].

**Table 1:** Characteristics of randomized controlled trials included in the meta-analysis.

Study [ref]	Study design/Jadad score	Patients No. (M/F);Age, mean (I/C)	Grade or FEV_1_%pred	Study group (n)	TC form or style	Protocol	Adheren/Adverse effects	Outcomes
Chan [19], [20]	Single-blind RCT/4	206 (188/18); 71.7/73.6	Class: I-III	TCQ (70); Exercise (69);Control (67)	13-form TCQ	12 wk × 2 times/wk;60 min/per time	83%/None	FEV_1_, FVC, 6 MWD, Dyspnea, HRQoL
Yeh [12]	Partial single-blind RCT/4	10 (6/4); 65.0/66.0	Mean class: 2.5	TC (5); Control (5)	Yang-style short form	12 wk × 2 times/wk;60 min/per time	91%/None	6 MWD, HRQoL
Leung [21]	Single-blind RCT/4	42 (27/15); Total agemean: 73.0	Total mean FEV_1_%pred: 59%	TC (22); Control (20)	Short-form Sun-style TC	12 wk × 2 times/wk;60 min/per time	91%/None	6 MWD, HRQoL, dyspnea
Li [27]	RCT/3	70 (55/15); 72.0/73.0	Class: II-III	TC (35); Control (35)	24-Short form TC	24 wk × 1 time/day;60 min/per time	86%/None	FEV_1_, FVC, HRQoL
Yao [28]	RCT/2	80 (45/35); 66.1/66.2	Class: II-III	TC (40); Control (40)	Chen-style short form	12 wk × 1 time/day;30 min/per time	100%/None	Dyspnea
Zhang [29]	RCT/2	90 (51/39); 62.0/62.2	Total mean FEV_1_% pred: 52%	TC (30); Exercise (30);Control (30)	24-Short form TC	48 wk × 1 time/day;30–60 min/per time	100%/None	FEV_1_, 6 MWD, HRQoL
Zhou [30]	RCT/2	46 (28/18); 72/73	Class: I-II	TC (23); Control (23)	24-Short form TC	16 wk × 5 times/day;40 min/per time	100%/None	FEV_1_, FVC

M/F, Male/Female; I/C, Intervention/Control; FEV_1_, forced expiratory volume in one second; RCT, randomized controlled trial; TCQ, Tai Chi Qigong; FVC, forced vital capacity; 6 MWD, 6-minute walking distance; HRQoL, Health-Related Quality of Life.

### The Primary Outcomes

**Figure 3:**
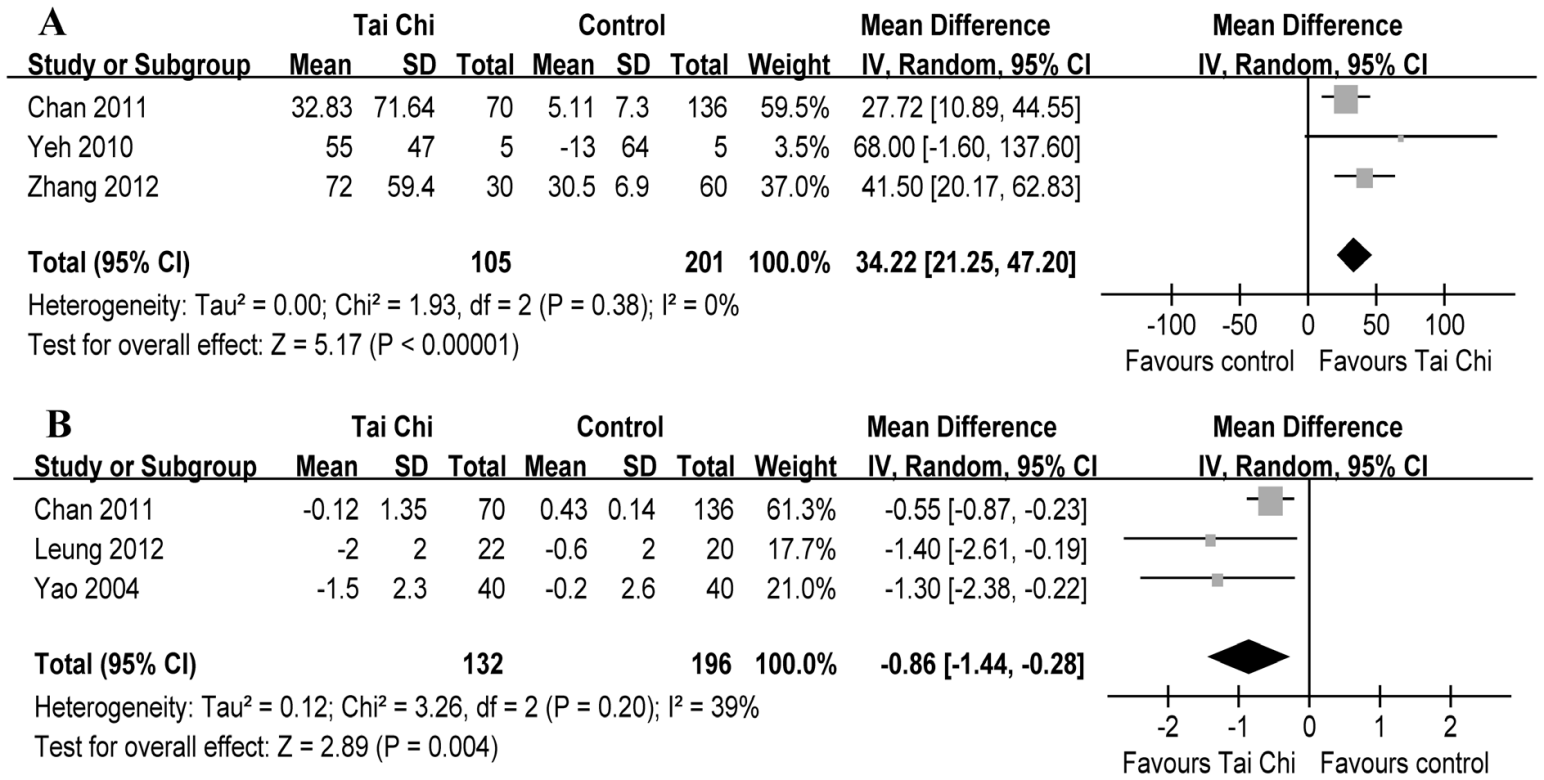
Meta-analysis of randomized controlled trials evaluating effects of Tai Chi on 6-min walking distance (A) and dyspnea (B) by the random-effects model.

The aggregate results of these studies suggested that TC was associated with a statistical improving on 6 MWD (3 RCTs, WMD 34.22 m, 95% CI 21.25–47.20, P<0.00001, P for heterogeneity = 0.38, I^2^ = 0%) [12], [19], [29] (Fig. 3-A) and on dyspnea (3 RCTs, WMD –0.86 units, 95% CI –1.44––0.28, P = 0.004, P for heterogeneity = 0.20, I^2^ = 39%) [19], [21], [28] (Fig. 3-B). Subsequently, the mean changes of 6 MWD were greater than the MCID (≥26 m) [31]; however, the mean changes of dyspnea were lower than the MCID of Borg scale (≥1 unit) [32].

Furthermore, we performed sensitivity analyses based on a random-effects model to explore the influence of the total effect according to methodological quality. Table 2 shows the results of sensitivity analyses excluding study with low quality for 6 MWD and dyspnea. However, these did not materially alter the overall combined WMD.

**Table 2:** Sensitivity analyses excluding trials with low quality for 6 MWD and dyspnea.

Outcome	n (N)	WMD (95% CI)	P value	I^2^ (%)	P_heterogeneity_
6 MWD					
All included trials [12], [19], [29]	306 (3)	34.22 (21.25–47.20)	<0.00001	0	0.38
High quality trials [12], [19] (Jadad score ≥3)	216 (2)	33.12 (6.22–60.03)	0.02	18	0.27
Low quality trial [29] (Jadad score <3)	90 (1)	41.50 (20.17–62.83)	0.0001	–	–
Dyspnea					
All included trials [19], [21], [28]	318 (3)	–0.86 (–1.44––0.28)	0.004	39	0.20
High quality trials [19], [21] (Jadad score ≥3)	248 (2)	–0.77 (–1.49––0.04)	0.004	44	0.18
Low quality trial [28] (Jadad score <3)	80 (1)	–1.30 (–2.38––0.22)	0.02	–	–

6 MWD, 6-minute walking distance; n, number of patients; N, number of trials.

### The Secondary Outcomes

**Figure 4:**
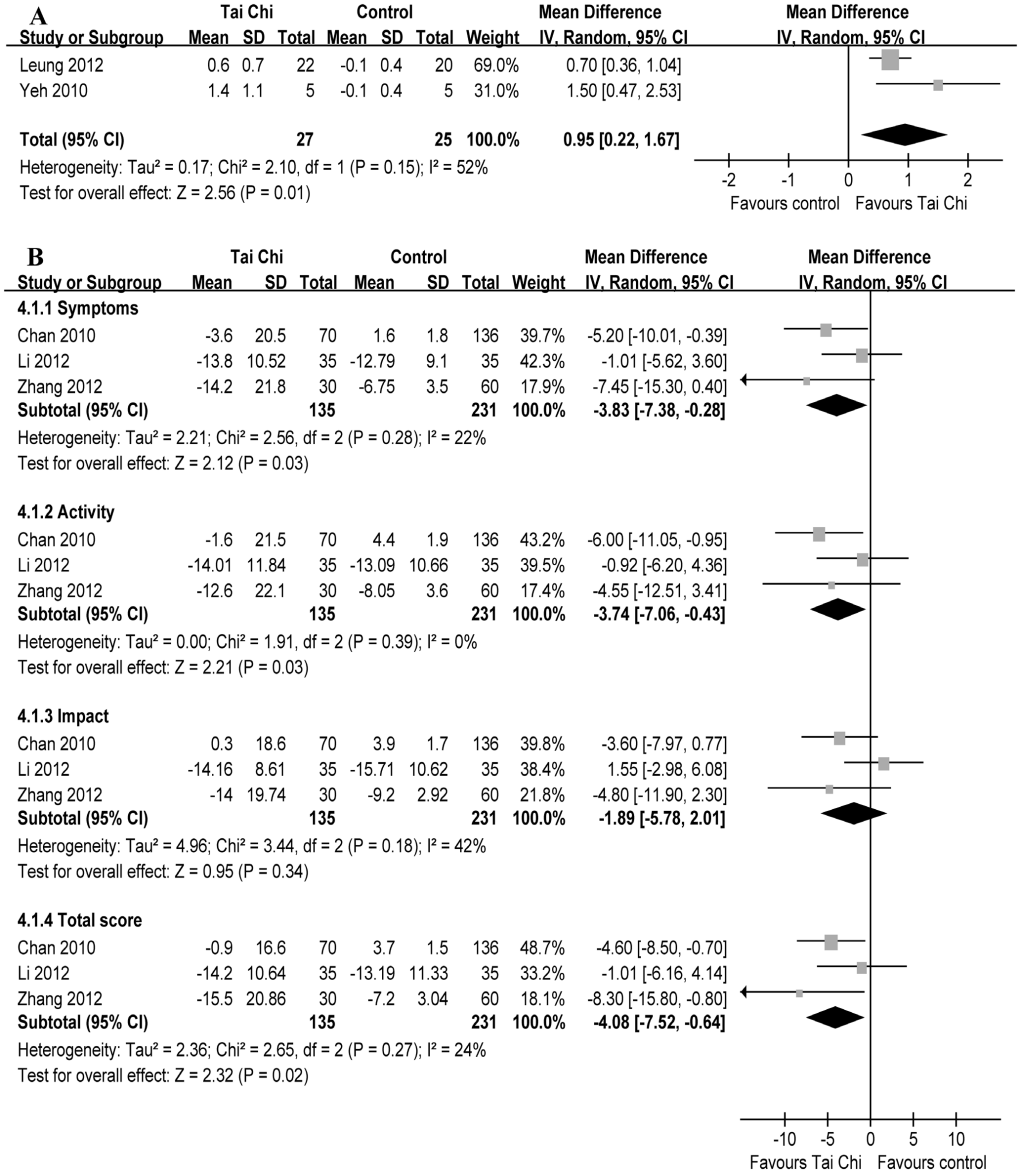
Meta-analysis of randomized controlled trials evaluating effects of Tai Chi on health-related quality of life by the random-effects model. Tai Chi was associated with a statistical improving on Chronic Respiratory Disease Questionnaire total score (A) and on St George’s Respiratory Questionnaire score except impact score (B).

The aggregate results of HRQoL suggested TC was associated with a statistical improving on the CRDQ total score (2 RCTs, WMD 0.95 scores, 95% CI 0.22–1.67, P = 0.01, P for heterogeneity = 0.15, I^2^ = 52%) [12], [21] (Fig. 4-A). The pooled WMDs from3 RCTs [20], [27], [29] for SGRQ were –4.08 scores (95% CI –7.52––0.64, P = 0.02, P for heterogeneity = 0.27, I^2^ = 24%) for total score, –3.83 scores (95% CI –7.38––0.28, P = 0.03, P for heterogeneity = 0.28, I^2^ = 22%) for symptoms score, –3.74 scores (95% CI –7.06––0.43, P = 0.03, P for heterogeneity = 0.39, I^2^ = 0%) for activity score, –1.89 scores (95% CI –5.78–2.01, P = 0.34, P for heterogeneity = 0.18, I^2^ = 42%) for impact score (Fig. 4-B). Subsequently, the changes of the CRDQ total score were greater than the MCID (≥0.5 scores) [33]; however, the changes of SGRQ were similar to or greater than the MCID (≥4 units) except impact score [34].

**Figure 5:**
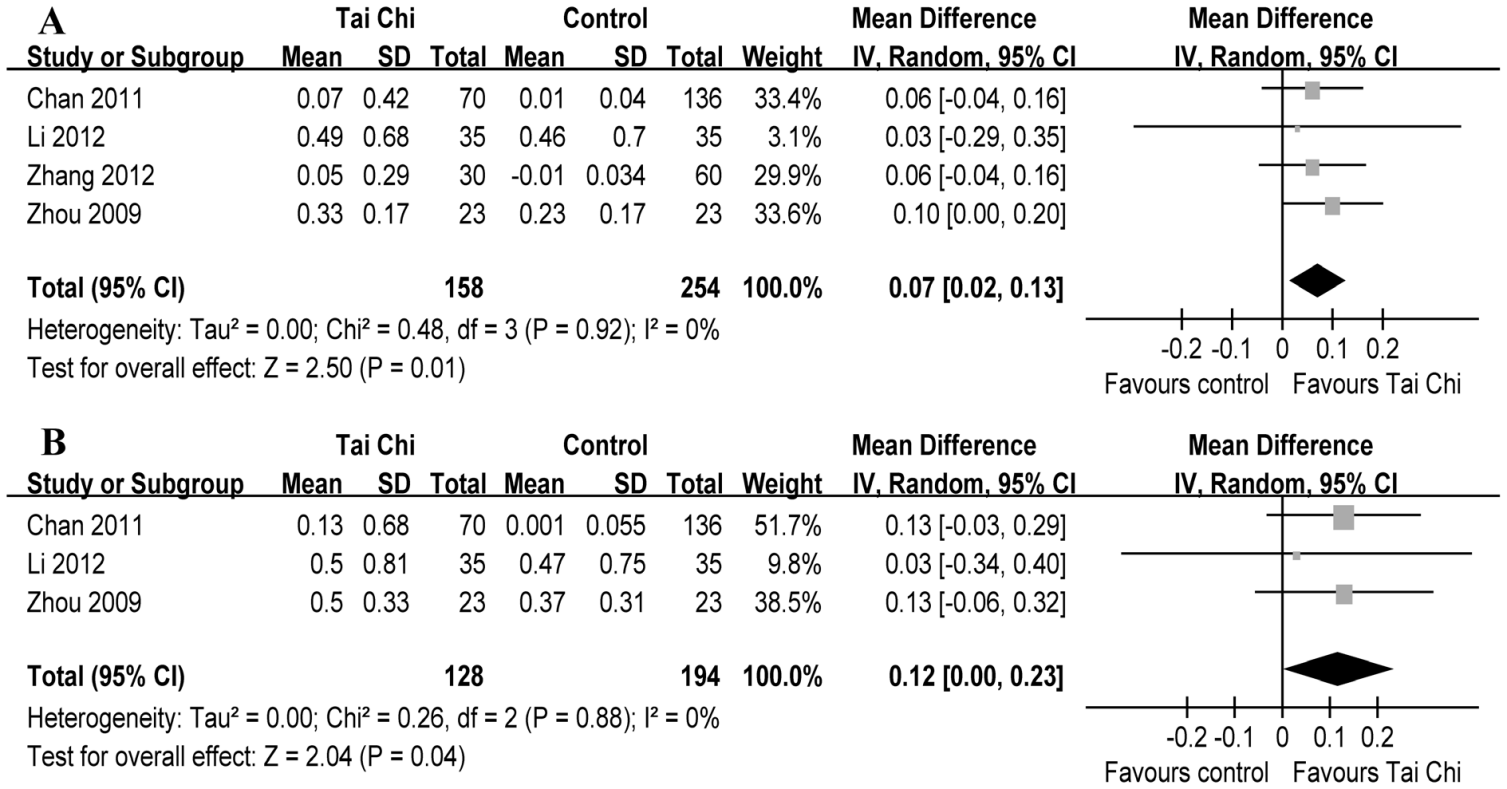
Meta-analysis of randomized controlled trials evaluating effects of Tai Chi on pre-bronchodilator spirometry by the random-effects model. Tai Chi statistically increased forced expiratory volume in one second (A) and forced vital capacity (B).

Regarding lung function, TC statistically increased FEV_1_ (4 RCTs, WMD 70 ml, 95% CI 0.02–0.13, P* = *0.01, P for heterogeneity = 0.92, I^2^ = 0%) [19], [27], [29], [30] (Fig. 5-A) and FVC (3 RCTs, WMD 120 ml, 95% CI 0.00–0.23, P = 0.04, P for heterogeneity = 0.88, I^2^ = 0%) [19], [27], [30] (Fig. 5-B). Subsequently, the changes of FEV_1_ were lower than the MCID (≥100 ml) [35].

### Adherence and Adverse Effects

None of all RCTs reported any side effects or exercise-related problems. Chan *et al.* reported increasing dyspnea and joint pain which were not related to TC program [19]. The TC program attendance rate ranged from 83% to 100% (Table 1).

## Discussion

The main purpose of the current meta-analysis is to evaluate the role of TC in COPD patients. The preliminary evidence suggested that TC may improve exercise capacity, dyspnea, quality of life, and lung function compared with general exercise control or routine care in COPD patients. However, there is currently a lack of adequate data and/or sufficient clinical evidence to support TC benefiting positive effects on these important clinical endpoints.

One of our results showed that TC was associated with statistical improvements regarding the outcomes included. However, it should be emphasized that the results were needed to compare with the MCID inasmuch as not all statistically significant differences are clinically relevant when interpreting clinical measures [36]. The changes of 34.22 m for 6 MWD were greater than the MCID (≥26 m) [31], which indicates that there should be a clinical effect for TC in COPD patients. However, there may be a moderate or even a slight effect with regard to 6 MWD for TC compared with other approaches such as upper limb and lower limb exercise training [9], [37]. Next, the changes of dyspnea were lower than the recommended MCID of Borg scale (≥1 unit) [32]. However, the changes of 0.86 units showed that there may be a better effect regarding to dyspnea for TC compared with upper extremity exercise [38]. Moreover, sensitivity analyses excluding low quality trial did not materially alter the pooled results, which adds robustness to our primary findings. In terms of HRQoL, the changes in CRDQ total score exceeded the MCID (≥0.5 scores) [33], which is consistent with the result from a research [21]. Unfortunately, we failed to further compare each domain of CRDQ with the MCID due to currently insufficient data. In addition, the changes in SGRQ were similar to or even greater than the MCID (≥4 units) except impact score [34]. Therefore, according to the findings, we believe that TC may improve HRQoL in patients with COPD. Of note, although imperfect with disease state, morbidity, mortality and functional status, FEV_1_ and FVC are the most commonly reported forced expiratory spirometry because of good reproducibility, ease of measurement, and correlation [35]. The changes of 70 ml for FEV_1_ were lower than the MCID (≥100 ml) [35]. Notwithstanding, taking into account the damaged lung function is difficult to reverse [6], the encouraging results showed that there may be a potential advantage for TC compared with other approaches of PRPs such as inspiratory muscle training, nutritional supplementation, education, etc [6].

Another concern with TC was adverse events and adherence. In view of the overall studies, no adverse events related to TC were reported. It is also worth noting TC adherence was very high, and that patient withdrawal in TC groups was very low, with most drop-out being due to hospitalization or communication barriers. These indicate TC appears to be generally safe and engenders good compliance among patients. Based on these findings, TC is well tolerated and enjoyed by the majority of the participants. Given that there are no special equipment requirements, lower costs and potential physical benefits, TC may represent an interesting alternative to conventional exercise training and incorporation into PRPs for COPD patients, and should be considered and worthy of further study.

Several limitations of the current meta-analysis should be taken into account. First, the number of studies is very limited and not more than 2–4 studies are available for the main outcomes, which limits the conclusion. Besides, these studies have wide variation in sample size. Overestimation of the treatment effect is more likely in smaller trials compared with larger trials. Second, different intervention forms represent probably one of the most important confounders. Particularly, for an intervention such as TC where heterogeneity is the standard, it becomes important to understand what was done. Style, emphasis, intensity, instructor characteristics all may make a substantial difference and greatly affect interpretation of results. Third, there was a certain risk of bias and heterogeneity among the included trials, especially among four RCTs from China with lack of adequate allocation concealment and blinding. The targeted population varied greatly (e.g., age, gender, ethnicity, GOLD class). Considering that mainly in patients that becuase of cultural or age related issues may be not so prone to be involved in TC training, these factors may contribute to the heterogeneity and have a potential influence on the results. Next, several caveats must be considered. Since there were limited data in analyzing each outcome and different measures to evaluate outcomes which may have a potentially negative impact, results should be interpreted with caution. Finally, some missing and unpublished data may lead to bias.

Nonetheless, the present study also provides additional interesting clues about a novel exercise to PRPs that may be useful for future research on this topic. Firstly, blinding prevents ascertainment bias and protects the sequence after allocation [39]. Although it may be difficult to blind to investigators, participants, and outcome assessors in TC study, we should try to apply the appropriate blinding such as blind outcome assessments. Secondly, it is crucial that PRPs tailor COPD patients; however, TC movements are far from “standard” and there is much variability. Exercise intensity will vary greatly depending on the fitness of the individual, the style, depth of stances, the vigour of performing the movements, etc. Further studies need to pay attention to the suitable?TC form for COPD patients. In addition, six to twelve weeks of PRPs benefit in several outcomes that decline gradually over 12 to 18 months in patients with COPD [6]. However, our study showed that the follow-up only ranged from 12 to 48 weeks; thus far the long-term effects of TC and the optimal exercise duration remain unclear. Next, it remains unknown which aspects of TC are responsible for the beneficial effects, and how TC differs from other forms of exercise (e.g., walking, upper limb and lower limb strength and endurance training, etc.). Finally, most studies lacked other objective outcome measures such as exacerbation rate, peripheral muscle strength, balance, survival and immune function, especially at the molecular level [40]. Therefore, further studies should focus on them to better understand the specific mechanism and obtain more reliable and convincing evidence for the efficacy of TC in patients with COPD.

In summary, the current encouraging evidence suggests that TC may improve exercise capacity, dyspnea, HRQoL, and lung function in COPD patients; thus, TC should be encouraged to be a potential approach to PRPs. However, considering the potential bias and heterogeneity of available data and the limited RCTs, further larger-scale RCTs are needed to substantiate the preliminary findings and investigate the long-term effects of TC as well as the tailoring of the rehabilitation intervention for COPD patients.
